# RAGE Inhibitors for Targeted Therapy of Cancer: A Comprehensive Review

**DOI:** 10.3390/ijms24010266

**Published:** 2022-12-23

**Authors:** Tabrez Faruqui, Mohd Sajid Khan, Yusuf Akhter, Salman Khan, Zeeshan Rafi, Mohd Saeed, Ihn Han, Eun-Ha Choi, Dharmendra Kumar Yadav

**Affiliations:** 1 Department of Biosciences, Integral University, Dasauli, P.O. Basha, Kursi Road, Lucknow 226026, Uttar Pradesh, India; 2 Department of Biochemistry, Aligarh Muslim University, Aligarh 202002, Uttar Pradesh, India; 3 Department of Biotechnology, Babasaheb Bhimrao Ambedkar University, Vidya Vihar, Raebareli Road, Lucknow 226025, Uttar Pradesh, India; 4 Plasma Bioscience Research Center, Applied Plasma Medicine Center, Department of Electrical & Biological Physics, Kwangwoon University, Seoul 01897, Republic of Korea; 5 Department of Biology, College of Sciences, University of Hail, Hail P.O. Box 2240, Saudi Arabia; 6 Gachon Institute of Pharmaceutical Science and Department of Pharmacy, College of Pharmacy, Gachon University, Hambakmoeiro 191, Yeonsu-gu, Incheon 21924, Republic of Korea

**Keywords:** cancer, RAGE, RAGE inhibitor, targeted therapy, end products

## Abstract

The receptor for advanced glycation end products (RAGE) is a member of the immunoglobulin family that is overexpressed in several cancers. RAGE is highly expressed in the lung, and its expression increases proportionally at the site of inflammation. This receptor can bind a variety of ligands, including advanced glycation end products, high mobility group box 1, S100 proteins, adhesion molecules, complement components, advanced lipoxidation end products, lipopolysaccharides, and other molecules that mediate cellular responses related to acute and chronic inflammation. RAGE serves as an important node for the initiation and stimulation of cell stress and growth signaling mechanisms that promote carcinogenesis, tumor propagation, and metastatic potential. In this review, we discuss different aspects of RAGE and its prominent ligands implicated in cancer pathogenesis and describe current findings that provide insights into the significant role played by RAGE in cancer. Cancer development can be hindered by inhibiting the interaction of RAGE with its ligands, and this could provide an effective strategy for cancer treatment.

## 1. Introduction

Cancer is a leading cause of mortality globally—it is estimated to have accounted for 10 million deaths in 2020, which is approximately one-sixth of all deaths worldwide. In 2020, the most prevalent cancers were lung, breast, colorectal, prostate, skin (nonmelanoma), and stomach cancers, with 2.21, 2.26, 1.93, 1.41, 1.2, and 1.09 million cases, respectively. The most prevalent causes of cancer-related deaths were lung, colorectal, stomach, liver, and breast cancers, which accounted for 1.80, 0.935, 0.769, 0.83, and 0.685 million deaths, respectively [1]. 

Receptor for advanced glycation end products (RAGE) is a 45 kDa multiligand transmembrane receptor in the immunoglobulin (Ig) superfamily that plays an important role in pathophysiological activities and has attracted considerable attention for therapeutic applications [2,3]. RAGE expression is highly downregulated in lung cancers, whereas its ligands are widely overexpressed. In rat lungs, its expression is gradually increased from the fetal stage until birth and is high in the mature stage. However, RAGE is either not expressed in non-small cell lung cancer (NSCLC) tissues or its expression is significantly reduced compared with that in normal lungs, leading to reduced RAGE levels, which contribute to the impairment of cells, differentiation, as well as arrangement of the epithelial structure, with associated oncogenic transformation. RAGE is thought to be associated with the development and invasiveness of lung tumors, as well as angiogenesis, and is thus assessed as a diagnostic marker for lung malignancy. However, Hsieh et al. reported the extensive expression of RAGE and its ligand, S100A6, in human lung cancer, suggesting a possible role of RAGE-mediated signaling in the development of this cancer [4,5,6,7,8,9,10]. Recent findings confirm that RAGE levels are increased in various malignancies, including breast, pancreatic, prostate, colorectal, gastric, and liver cancer [2,11,12,13,14]. Because of its ability to discriminate between a wide range of structurally distinct endogenous and exogenous ligands, RAGE is classified as a pattern recognition receptor (PRR) and damaged-associated molecular pattern (DAMP) [15]. RAGE is present at moderate concentrations in most organs, except the lung, where it is highly expressed in alveolar (AT-1) epithelial cells [16]. RAGE can interact with multiple ligands, including AGEs, high-mobility group box1 (HMGB1), S100/Calgranulins family members, β-sheet fibrils, prions, adhesion molecules, complement components, advanced lipoxidation end products (ALEs), lipopolysaccharide, and others [17,18].

In identical tumor types, increased RAGE expression has been associated with increased tumor histological progression [10,13,19,20,21,22]. In vitro and in vivo experiments revealed that deletion of the short cytoplasmic domain of RAGE exerts a dominant-negative effect, impairing the signal transduction response to the RAGE ligand. The cytoplasmic RAGE domain is strongly charged and is critical for signaling and activation. Ligand–RAGE interaction stimulates cellular transport and many signal transduction cascades, including mitogen-activated protein kinases, phosphatidylinositol 3-kinase, Jak/STAT, and, most notably, the Rho GTPases, Rac-1 and Cdc-42 [11,23,24,25,26,27,28,29]. The expression of RAGE has been shown in a variety of human and mouse tumors, including pancreatic, breast, and colonic carcinomas, fibrosarcoma, and melanoma cell lines. A murine model with high RAGE expression has also been reported. RAGE and its ligand, HMGB1, were also found to be expressed in a murine model [30]. Recent studies suggest that RAGE–ligand interactions affect cellular responses, such as proliferation, survival, migration, and metastasis by activating MAP kinase (Erk1/2), Ras-extracellular signal-regulated kinase, Cdc42/Rac, stress-activated protein kinase/c-Jun-NH2–terminal kinase, and p38 mitogen-activated protein kinase [11,14,20,23,24,27,30,31]. RAGE also induces an NF-κB response. NF-κB is a transcription factor that protects against apoptosis, inducing antiapoptotic genes, such as A1, A20, XIAP, Bcl-2, and Bcl-XL, which are crucial for cell survival under constant cellular stimulation [32,33].

**Figure 1:**
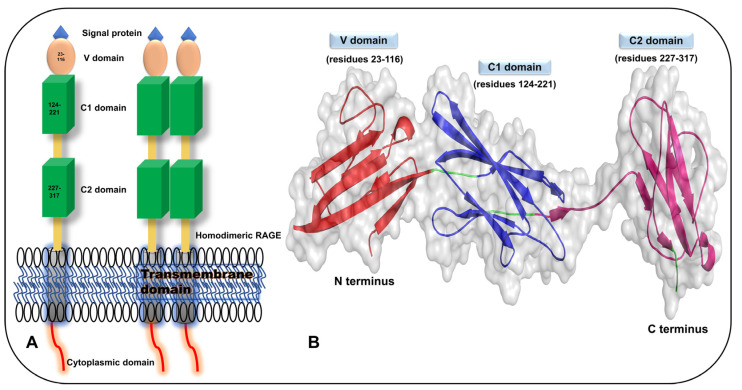
(**A**) Head-to-toe organization of receptor for advanced glycation end products (RAGE), consisting of a signal protein (residues 1–22), extracellular domains (V (residues 23–116), C1 (residues 124–221), and C2 (residues 227–317), a transmembrane domain (residues 343–363), and a cytosolic domain (residues 364–404); and (**B**) a hypothetical model of the extracellular region of RAGE with V, C1, and C2 domains.

Full-length RAGE comprises a signal peptide (residues 1–22), an extracellular domain (residues 23–342) flanked by V, C1, and C2 domains, a transmembrane domain (residues 343–363), and a cytoplasmic domain (residues 364–404) as shown in Figure 1A,B. The C1 domain has basic amino acids on the surface, whereas the C2 domain is negatively charged, which offers the ability for dimerization of RAGE [34]. Among the RAGE domains, Gln119 in the V domain is implicated in beta-sheet H-bonding with Tyr150 on the C1 domain, exhibiting hydrophobicity between H-bonds. In addition, in the C1 domain, an H_2_O molecule connects to the amide nitrogen of Ile120 via the backbone atoms of Glu119 and Arg29 in the V domain. As hydrophobic interactions occur between the backbone atoms of Pro215 via the C1 F-G loop and Tyr118 via the C1 B-C loop and Ile91, the V and C1 domains of RAGE are consistently conserved and the VC1 domains have the same components [35,36]. The V and C1 domains, which form an integrated unit, VC1, are involved in the binding of RAGE with its ligands; however, the C1 domain also plays an important role in the identification of ligands [35,36,37,38]. Binding to RAGE involves homodimerization or oligomerization, characterized by different mechanisms that involve amino acid residues in the V and transmembrane domains [36,39]. Because RAGE monomer shows poor binding with monomeric ligands, binding to RAGE requires its oligomerization, which involves high-affinity binding between the receptor and ligand. Oligomerization of RAGE is mediated by the self-association of C1-C1 and C2-C2 domains [35,40,41].

**Figure 2:**
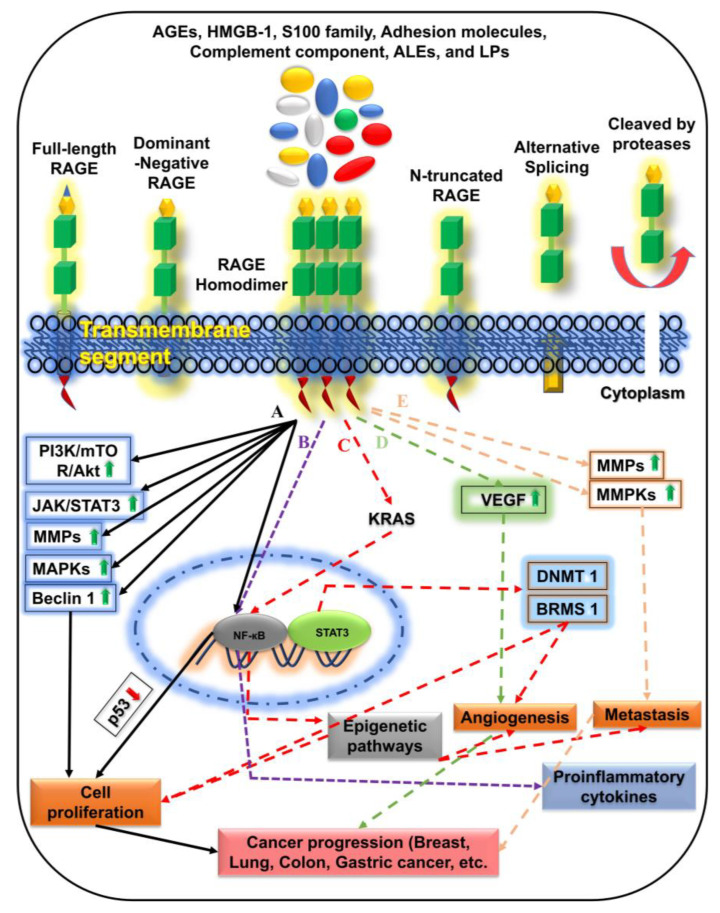
Schematic illustration of the functions of a receptor for advanced glycation end products (RAGE) in the cancer microenvironment: (**A**) binding of RAGE and its ligands promotes angiogenesis and cancer development by upregulating PKI3/mTOR/Akt, MAPKs, MMPs, VEGF, NF-κB, and downregulating p53 signaling; (**B**) secretion of HMGB1 from inflamed or injured cells followed by its binding with RAGE results in the increased release of proinflammatory cytokines via NF-κB activation and ensuing harmful inflammatory responses; (**C**) HMGB1 operates via NF-κB and epigenetic pathways in the cancer microenvironment to exert its long-lasting effects on surviving tumor cells, immune cells, and stromal cells. Together with STAT3, NF-κB controls genes that promote metastasis, angiogenesis, and cancer development; (**D**) upregulation of VEGF leads to angiogenesis resulting in cancer progression; and (**E**) increased expression of MMPs and MMPKs promotes metastasis in various cancers.

Different isoforms of RAGE, including full-length RAGE, dominant-negative RAGE, N-truncated RAGE, and C-truncated secretory/soluble RAGE, have been reported. Excessive accumulation of RAGE on the cell membrane leads to multiple stimulatory and immunological responses [42,43,44]. RAGE expression is high in vascular and cancer cells. It binds to different ligands including calgranulin family proteins, HMGB1, amyloid β-peptide, β-sheet fibrils, advanced oxidation products (AOPs), Mac-1, and phosphatidylserine. The binding of these ligands to RAGE stimulates MAPK and NF-κB, which induces cellular proliferation as shown in Figure 2A [23,26,45,46,47,48,49]. Moreover, excessive RAGE expression prevents cell death under hypoxia and chemotherapy in cancer cells [27,50]. Studies on diabetes mellitus and hyperglycemia reveal a connection with breast cancer. RAGE, as a PRR, is implicated in inflammatory diseases, and its stimulation in concert with diabetes contributes to the progression of cancer [51,52,53].

Calgranulins are small cell signaling proteins with Ca-binding sites and EF-hand motifs. These S100 family members act as stimulatory ligands that ensure interaction between two or more monomers for intracellular and extracellular processes and are responsible for cell growth, differentiation, and mobility [2,54,55]. 

The shortage of oxygen in tumors necessitates the glycolytic metabolism of glucose to fulfill the energy needs and is associated with the increased formation of advanced glycation end products (AGEs). Carboxymethyllysine (CML) is a lysine glycation end product that has been identified as one of the most prominent AGEs of tissue proteins. RAGE is a receptor for AGEs in various human malignancies; nonetheless, the presence of AGEs in human malignancies has largely been unexplored [26,45,56,57].

Amphoterin (HMGB1) is an electrically neutral non-histone protein involved in transcription, DNA repair, differentiation, neural development, and extracellular signaling, and has a plausible connection with cancer. HMGB1 is a cytokine released by monocytes and macrophages or other cells, except for apoptotic cells. It is responsible for the pathogenesis of various inflammatory diseases and is involved in the secretion of inflammatory cytokines [10,22,58,59]. The interaction of RAGE/TLRs with HMGB1 is associated with the growth, progression, and metastasis of malignant tumors [60,61]. The binding of HMGB1 with RAGE promotes chemotaxis and the secretion of cytokines, which corresponds with the activation of NF-κB [62,63]. Because RAGE and its ligands are implicated in many malignancies, small molecules that target RAGE and its ligands may help treat various tumors. 

In the present review, we discussed various aspects of RAGE and its prominent ligands, which are implicated in cancer pathogenesis, and summarized the recent findings that will provide novel insights into the significant role of RAGE and its ligands in cancer.

## 2. Immunotherapy for Cancer Treatment

Immunotherapy, in which immune cells are stimulated to destroy cancer cells, has gained significant attention among researchers and physicians as a valuable therapeutic option besides chemotherapy and targeted therapy. Exciting clinical remissions have been reported in incurably ill cancer patients with no other treatment options, which have laid the framework for many innovative cancer immunotherapies that are presently being investigated in clinical trials. However, the treatment outcomes of this remarkable approach are only relevant in 20–30% of all cancers [64,65,66]. Immunotherapy, often known as biological therapy, is a type of cancer treatment that involves the use of chemicals or molecules derived from living organisms (e.g., cancer vaccines, checkpoint inhibitors, immune system modulators, monoclonal antibodies, and T cells Lymphoma). Checkpoint inhibitors are increasingly being used in immunotherapy [67,68]. RAGE and its ligands are located on the surface of cells, including tumor cells and immune cells, providing avenues for cancer therapy using specific inhibitors.

## 3. Carbonyl Stress and RAGE Ligands in Cancer

### 3.1. Advanced Glycation End Products (AGEs)

AGEs are products of nonenzymatic glycation, in which the bases of DNA react with reducing sugars, such as glucose, fructose, or deoxyribose, to form a Schiff’s base, which can lead to the formation of an Amadori product through an enamine intermediate. Furthermore, AGEs can also be formed through a variety of different processes, such as the oxidation of sugars, lipids, and amino acids, which produce active aldehydes that eventually become AGEs. The Amadori product undergoes dehydration, cyclization, condensation, and isomerization, culminating in the production of AGEs [69]. The formation of AGEs is typically associated with increased free radical activity. AGEs can modify cell membranes and account for gene alterations leading to the malignant transformation of cells [70]. N-carboxyethyllysine (CEL), N-carboxymethyllysine (CML), methylglyoxal lysine dimer (MOLD), glyoxal lysine dimer (GOLD), and pentosidine are some of the AGEs that have been identified. Regardless of their different nature or the state of cross-links, different AGEs are known to be important in the development of a variety of diseases, including cancer [71]. Diabetes is defined by elevated oxidative stress conditions. Experimental data have demonstrated an increased risk for numerous types of cancers in diabetic patients. Hyperglycemia promotes oxidative stress in various cells through numerous metabolic pathways and causes oxidative DNA damage, which is a precursor to carcinogenesis. 

Accumulating evidence implicates AGEs in aging, diabetes, and cancer. The interaction of RAGE with AGEs causes oxidative stress, which in turn promotes the production of AGEs and increases their interaction with RAGE [72]. RAGE–AGE interaction promotes the activation of the PI3K, oncogenic Ras, PKC, and Rho/GTPase (Cdc42 and Rac-1) signaling pathways shown in Figure 2, which leads to cell growth, stress responses, apoptosis, the release of growth factors and proinflammatory cytokines, and motility via changes in cellular properties [73,74]. Clinical evidence shows that individuals with oral cancer who also have diabetes mellitus have a higher risk of cancer spread and have lower survival rates [75]. The binding of RAGE with AGEs in human oral cancer cells is associated with metastasis and angiogenesis [75]. The interaction results in oxidative and glycation stress and its products may have life-threatening repercussions in the lung, oral, prostate, and breast cancers. Pathways, such as p38 MAPK, NF-κB, TNF-α, and AGE–RAGE interactions, may play a vital role in cell growth, propagation, apoptosis, and metastasis [76]. Treatment options remain insufficient for many tumors. We perceived certain important pathways that may play a key role as therapeutic targets in diverse types of malignancies.

### 3.2. High-Mobility Group Box1

HMGB1 is made up of an N-terminal A segment (residues 1–83), a B segment (residues 88–164), and a C-terminal acidic tail [77]. Receptors for HMGB1—RAGE and Toll-like receptors (TLRs)—exist on the surface of white blood cells as well as on endothelial cells; RAGE and TLRs activate the expression of NF-κB, which is responsible for the activation of genes encoding adhesion proteins as well as inflammatory cytokines and proangiogenic factors. HMGB1 influences the maturation and progression of liver, breast, colon, and gastrointestinal cancers [50,78,79,80,81,82,83]. It regulates the MAPK pathway in gastric, renal, colon, and liver carcinogenesis in mice. HMGB1 is primarily found in the nucleus, although it can translocate to the cytoplasm in response to cellular stress and injury. Through interaction with Bcl-1 and dissociation from Bcl-2, it regulates autophagy and apoptosis [21,79,84,85,86,87].

In vitro and in vivo studies revealed that HMBG-1 promotes PGDF activity and expression of the vascular endothelial factor. The expression of HMGB1 involves various autocrine/paracrine factors, which are responsible for the positive feedback mechanism to its receptors. HMGB1 stimulates and promotes the expression of proangiogenic genes in endothelial cells. It may bind various receptors, including RAGE, TLRs, and syndecan-1 (CD138), which activate NF-κB and ERK1/2 (Figure 2C). It can also bind lipid molecules, such as sulfatide and phosphatidylserine lipids, and can react with lipopolysaccharides and modify CD14 via TLR4-mediated signaling.

Extracellular HMGB1 interacts with RAGE to increase the release of proinflammatory cytokines via NF-κB activation, which is responsible for harmful inflammatory responses [88,89,90,91,92].

The binding of HMGB1 with RAGE through its carboxyl-terminal tail can stimulate RAGE, a criterion for cell motility; its binding to RAGE has a synergistic effect with a dominant inhibitor in stopping the progression of cancer [93]. HMGB1 is characterized by two homologous HMG-boxes, box A and box B, which are linked through linker proteins, approximately 30 and 20 amino acids long. HMGB1 acts as a chaperone to facilitate the incredibly quick binding of transcription factors to DNA [23,94,95]. It plays a very important role in the binding of the tumor suppressor protein, p53, to DNA. HMGB1 decreases p53 expression, which causes DNA damage [94,96,97]. HMGB1 binds to the V domain of RAGE, driving dynamic protein concatenation as well as cell migration via GTPases, such as Cdc42 and Rac; the RAGE binding site is in the HMGB1 box B at amino acid residues 150–183 [98]. In vitro, the binding of HMGB1 with RAGE and its effects through NF-κB have been documented in numerous types of cancers, including human pancreatic cancer (BxPC-3), human hepatocarcinoma (HUH7, H22, HCC), human fibrosarcoma (HT1080), human NSCLC, human bladder carcinoma (5637, BIU (Colo320 and WiDr)), human renal cell carcinoma (CCRCC), murine lung cancer (Lewis cells), mice hepatocarcinoma, and human gastric carcinoma (BGC-823, SGC-7901, MKN-28, and MKN-45); its effects mediated through Bcl-2 have been reported in mouse neuroblastoma (Neuro2a), human neuroblastoma (SH-SY5Y), and human nasopharynges (MCF-7) [11,78,80,84,85,86,99,100,101,102,103,104,105,106,107,108,109,110,111,112,113].

### 3.3. S100/Calgranulin Family of Proteins

Ca^2+^ is a second messenger that regulates nerve conduction, muscle contraction, cell motility, gene expression, cell mortality, and necrosis. Ca^2+^ signaling is controlled by calcium-binding proteins, which regulate Ca^2+^ concentration in the cytoplasm [87,114,115,116]. S100 proteins are a family of calcium-binding proteins, which are involved in calcium homeostasis [117]. In addition, S100 proteins also bind Zn^2+^. S100 proteins, such as S100B, S100A6, S100A7, S100A8, S100A9, S100A10, and S100A12, are EF-hand calcium-binding proteins that form homo- and heterodimers. Cysteine and histidine are major constituent amino acids in these proteins. These proteins undergo a structural change upon calcium-binding, which is responsible for protein–protein interaction. The protein–protein interaction site of S100B contains hydrophobic and polar residues required for high binding affinity. S100B functions extracellularly in a cytokine-like manner and shows unique interaction with RAGE [55,118,119,120].

#### 3.3.1. S100A4/Calvasculin

S100A4, also known as calvasculin/metastatin/placental calcium-binding protein/protein Mts1, is abundantly expressed in various cancers. Its expression is associated with malignant growth. It affects various cellular processes, such as angiogenesis, metastasis, and cell growth, and also increases the expression of matrix metalloproteinases [121,122,123,124]. The binding of S100A4 with RAGE increases MAPK/ERK and hypoxia signaling [123,124,125]. In vitro, the activation of NF-κB upon the interaction of S100A4 with RAGE has been reported in various cancer cells, including human osteosarcoma (II-11b), human melanoma (A375, B16-F10), and human pancreatic cancer (BxPC-3) cells [110,126,127,128,129]. The expression of S100A4 is increased in breast cancer in humans, rats, and mice and metastatic cells compared with that in nonmetastatic cells [130,131]. Stimulation of II-11b cells by extracellular S100A4 results in increased NF-κB and JNK activation. S100A4 was shown to stimulate the expression of NF-κB, ERK/2, p38MAP kinase, and JNK in other cell systems. The binding of S100A4 with RAGE was reported to stimulate NF-κB, resulting in an increase in MMP-13 expression in chondrocytes. Also, through its NF-κB promoting activity, S100A4 activates IKK, following its binding to RAGE. S100A4 can promote angiogenesis via annexin II, and through interactions with heparan sulfate proteoglycans, it promotes neurite outgrowth. S100A4 can interact with various receptors to transmit varied biological information [124,132,133,134,135,136,137].

S100A4 has a 12-amino acid calcium-binding domain at the C-terminus and an N-terminal pseudo-EF-hand with 14 residues [127,138]. It is a homodimer with noncovalent interactions between helices 1 and 4, and is responsible for the X-type four-helix bundle; both subunits have two EF-hand calcium-binding domains that have an N-terminal pseudo-EF hand and a C-terminal canonical characteristic EF-hand with a small two-stranded antiparallel β-sheet [139,140,141]. S100A4 undergoes a significant conformational change in the canonical EF-hand upon calcium binding, resulting in the exposure of the hydrophobic binding pocket composed of helices 3, and 4, the hinge region (loop 2), and the C-terminal loop region. In two crystal structures of calcium-bound S100A4, the hydrophobic cleft of each subunit is occupied by the C terminus of an attached dimer, which is responsible for the formation of S100A4 oligomers that were detected extracellularly [142]. The calcium-binding site of S100A4 is located at the C-terminal end of helix 1, and Ca^2+^ is coordinated via the backbone carboxyl group at Ser20, Glu23, Asp25, Lys28, the side-chain carbonyl of Glu33, and an H_2_O molecule. The two binding sites are contiguous in space and involve hydrophobic interactions between Lys28 from site 1 and Glu69 from site 2 [125,135,140,141,142].

#### 3.3.2. S100A6/Calcyclin

Homodimeric S100A6 (10–11 kDa) is a calcium-binding protein that is believed to also interact with Zn^2+^. S100A6 belongs to the calgranulin family and is involved in the propagation of calcium signals. It is conspicuously located in the cytoplasm although it has also been detected in the extracellular matrix and various body fluids. It is highly expressed in the muscles, lungs, kidneys, and brain. The signaling pathway is aided by the extracellular binding of S100A6 with RAGE. Calcium binding induces a conformational change in S100A6, which increases its hydrophobicity and allows for interaction with target proteins. Elevated levels of S100A6 have been observed in epithelial cells, fibroblasts, and several types of tumor cells. The function of S100A6 is unknown; however, it is thought to be involved in cell propagation, cytoskeletal dynamics, and tumorigenesis [25,143,144,145,146].

**Figure 3:**
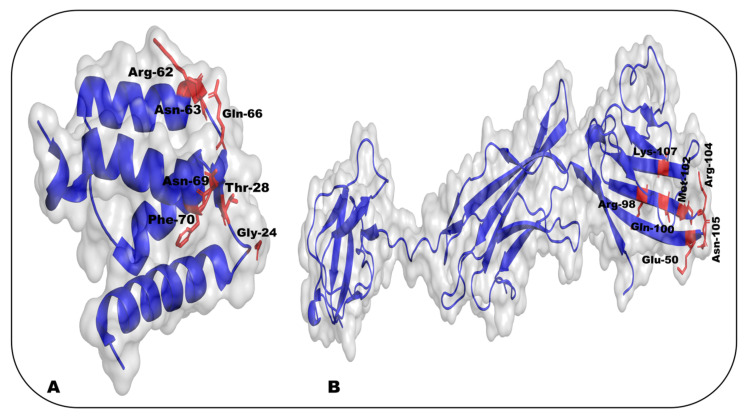
Three-dimensional representation of S100A6 and receptor for advanced glycation end products (RAGE) interacting amino acid residues: (**A**) S100A6 residues that interact with RAGE; and (**B**) residues in the V-domain of RAGE that interact with S100A6.

S100A6 functions as a homodimer formed by noncovalent interactions and binds two Ca^2+^ per monomer, each through an EF-hand structure composed of two helices connected by a short loop region. S100A6 can also bind two Zn^2+^ per monomer and can serve as a Zn^2+^ chelator mostly in the cytoplasm, but also outside the cell. Under conditions of increased Ca^2+^ concentration, S100A6 can associate with the nuclear envelope and plasma membrane [147,148]. The role of S100A6 signaling in breast cancer has previously been reported; it promotes the function of cacy/SIP, which is related to tumor invasion and metastasis by increasing β-catenin levels. In contrast, S100A6 levels are decreased in human breast cancer cell lines [143,149,150]. The binding of S100A6 with the V and C2 domains of recombinant RAGE has been reported. Studies have shown that the C1 and C2 segments, as well as their associated linker, configure a binding site for S100A6. Isothermal titration calorimetry for the binding of mutant S100A6 with the V domain of RAGE revealed the involvement of basic charged amino acids, Lys44, Arg48, Arg98, Arg104, and Lys107, and Met102, and Gly106, which form a hydrophobic region. Gly47, Leu49, Glu50, Gln100, Asn103, and Asn105 interact with mutant S100A6, allowing proximity of the basic amino acids to adjacent surfaces on the V domain of RAGE. Gln24, Thr28, Arg62, Asn63, Lys64, Gln66, Asn69, and Phe70 are the S100A6 amino acid residues in the protein–protein interface as shown in Figure 3A,B [151,152]. S100A6 is overexpressed in breast, stomach, and pancreatic cancers, as well as in thyroid carcinoma, clear cell renal cell carcinoma, and mixed lineage leukemia. Its expression is low in prostate and oral cancer, and it is used as a diagnostic or predictive marker in pancreatic, gastric, and prostate cancers, melanoma, NSCLC, and hepatocellular carcinoma [54,153,154,155,156,157,158].

#### 3.3.3. S100A7/Psoriasin

S100A7/psoriasin is a 12 kDa EF-hand calcium-binding protein in the S100 family that promotes cell migration through RAGE in a zinc-dependent manner. Extracellular expression of S100A7 is primarily associated with squamous cell cancer subtypes, namely lung cancer, head and neck cancer, cervical cancer, and bladder cancer, as well as with nonsquamous cancer subtypes, such as melanoma, breast cancer, and gastric cancer. S100A7, like S100A6, occurs in a homodimeric form with two Ca^2+^ EF-hand motifs and two Zn^2+^ ions at the binding site that span the dimer interface. S100A7 has been postulated to be involved in cell propagation, relocation, invasion, and tumor metastasis. Its expression is higher in cervical cancer compared with that in normal cervical tissues. The binding of S100A7 with RAGE activates the ERK signaling cascade, promotes cell mesenchymal competence, and induces epithelial–mesenchymal transition. Human S100A7 induces IL-1α expression in keratinocytes through RAGE-p38 MAPK-calpain-1 signaling (Figure 1A,B), and psoriasis-associated cytokines, such as IL-17a, IL-22, and IL-36, may upregulate S100A7 expression in keratinocytes [159,160,161,162,163,164,165,166,167,168,169,170]. Each monomer of the homodimer begins with helix I (Gln4 to Lys18), which leads through the first loop (Tyr19 to Asp27) into helix II (Lys28 to Phe39) where Pro40 results in a breach in the systematic helical pattern and a small turn linker helix II′ (Asn41 to Lys48) ensues in a different direction; this is followed by helix III (Tyr53 to Lys61), the calcium-binding loop (Asp62 to Asp72), and helix IV (Phe71 to Gln88). The zinc-binding site on S100A7 is mostly comprised of two histidines, His86 and His90, in the C terminus, and is located next to the end of helix IV [170].

#### 3.3.4. S100A8/Calgranulin-A and S100A9/Calgranulin-B

Cytosolic calcium regulates various cellular functions. Notably, calcium-binding proteins are important molecules in signal transduction, differentiation, and cell cycle regulation. S100A8/CACA/CFAG/MRP8 (10.8 kDa) and S100A9/MRP14 (13.2 kDa) are calcium-binding proteins expressed by neutrophils and activated monocytes. At basal concentrations, S100A8 and S100A9 display growth-promoting effects upon binding RAGE. Their binding to RAGE is implicated in breast cancer metastasis and stimulates the phosphorylation of LIN-11, IsL-1, and MEC-3 protein domain kinase, as well as cofilin. The binding of S100A8 and S100A9 with RAGE stabilizes Snail via NF-κB, enhances lung metastasis, and improves mesenchymal properties [171,172,173]. In vitro and in vivo data reveal homodimerization of S100A8 and S100A9, forming calprotectin; the resulting isoform may play a variety of roles in phagocyte physiology. S100A8 and S100A9 are found in the cytoplasm of neutrophils, monocyte membranes, plasma, and other body fluids. Calprotectin expression is associated with rheumatoid arthritis, autoimmune diseases, cystic fibrosis, chronic bronchitis, acute allograft rejection, gut irritation, inflammatory dermatoses, and abscesses, and it is a relevant marker for medical diagnosis [174,175,176,177,178]. RAGE is a key receptor of S100A8 and S100A9 in tumor cells, whereas TLR4 is a major receptor for both these ligands in macrophages.

Carboxylated N-glycans are expressed on RAGE, for example, on immune and tumor cells, which mediate S100A8/A9 and RAGE binding, promoting receptor-mediated signaling and pathogenesis. In colon cancer, S100A8 and S100A9 induce RAGE- and carboxylated glycan-dependent phosphorylation of ERK1/2 and SAPK/JNK, but in prostate and breast cancer, they promote p38 phosphorylation. The binding of S100A8/S100A9 on RAGE and carboxylated glycan in colon cancer activates the NF-κB pathways, resulting in a life-threatening link between inflammation and cancer. S100A8 and S100A9 are recognized as novel target genes in hepatic carcinoma cells during inflammation-mediated liver carcinogenesis [179,180,181,182,183,184,185]. Individually, S100A8 and S100A9 domains for the calcium-binding loop are fully conserved, and Ca^2+^ binds to the EF-hand I, which exhibits additional varied sequences correspondingly, as in the crystal structures with specific ligands. In the homodimers of S100A8 and S100A9 with calcium-binding sites at the N terminus occupied, each monomer is held together by a hydrophobic core of conserved sequences. Homodimers are stabilized by interactions of hydrophobic sidechains among these helices. S100A8 homodimer interaction takes place at the surface of each monomer between Leu72 and Ile76, whereas S100A9 interaction takes place on S100A8 at the site of Ile76 in a single chain that connects two helices. S100A9/S100A8 connection provides extra balance to the S100A9 subunit and promotes transition for a stretch of 6–14 amino acid residues at the C-terminal end, α-helix customs a disorganized state to α-helical configuration through increased electron compactness. S100A8 and S100A9 heterodimers have disulfide bridges between S100A9 at Asp30, and in S100A8 at His83 and His87, these amino acids participate in the binding of two Zn^+^ ions [186,187,188,189].

#### 3.3.5. S100A14/S100 Calcium-Binding Protein A14

S100A14 is an 11.6 kDa protein belonging to the S100 family. It has an N-glycosylation site, a protein kinase phosphorylation site, and an N-myristoylation motif [190]. S100A14 is highly expressed in some types of cancers, including ovarian, breast, and uterine cancers, and at low levels in the kidney, colon, and rectal cancers [191,192]. The binding of extracellular S100A14 with RAGE has been shown to control proliferation and apoptosis in esophageal cancer cells, and cell proliferation and invasion in oral squamous cell carcinoma [193,194,195]. Although the expression level and living purposes retain a tissue or malignancy character, S100A14 has been reported to remain contrary expressed in several human melanomas responsible for diverse life progression including propagation, apoptosis, cell motility keratinocytes variation, and motion transduction. S100A14 triggers the MAPK pathway, which causes lung adenocarcinoma, epithelial-mesenchymal transition cervical cancer cells, and metastatic breast cancer cells to proliferate. The interaction of RAGE and S100A14 is associated with increased cancer growth. However, S100A14 has been found to hinder gastric malignancy metastasis by hindering Ca^2+^ entry by limiting the FAK signaling pathway and MMP expression. In vivo and in vitro data demonstrate that S10014 inhibits NPC cellular motility via the NF-κB signaling pathway; moreover, it has been discovered that overexpression of S100A14 lowers the risk of NPC resistance to cisplatin [192,196,197,198,199,200]. Although the involvement of S100A14 in the translocation from the cytosol to the plasma membrane in breast cancer is well known, S100A14 has also been discovered to play a dynamic role in bladder cancer and growth. S100A14-related genes may be useful for detecting cancer in the peripheral blood of patients with advanced cancer. In vivo data reveal that the S100A14 gene is regulated by p53, which is associated with esophageal squamous cell cancer. Exogenous S100A14 ligation with RAGE on ESCC cell lines reported that a low dosage of exogenous S100A14 activates ERK1/2 and NF-κB signaling, stimulating cell proliferation or increasing cell endurance, as shown in Figure 1A.

On other hand, a higher amount of S100A14, can cause apoptosis and increase the generation of reactive oxygen species. Exogenous S100A14 stimulates cell proliferation or promotes apoptosis at different doses based on in vitro findings [201,202,203,204,205,206]. An S100A14 monomer interacts with aromatic amino acids in helix I at position Phe29 and in helix IV at positions Phe80, and Phe84, and additionally Trp85, which aids in helical orientation and maintains contact between two subunits. Numerous hydrophobic residues make the monomer–monomer on the helix I connection through helix I_0_ and helix IV_0_, and the various connection link residues in helices IV and IV_0_ become constant. The association between two monomers was intended to be enhanced by residues 53–57 in the hinge loop, which was recognized by residues at the N-terminal of helix I_0_ and the C-terminal segment of helix IV. As a result, it is said that S100A14 takes a conformation distinguished from that of the canonical apo arrangement, and that the Ca^2+^ loaded from helices II and III are almost antiparallel, employing the canonical apo arrangement of S100 proteins. However, the superimposition of the current structure of S100A13 with the structures of the apo and holo forms of S100A13 reveals a well-structured fold that used the last N-terminal of S100A14 with 13 residues for the Ca^2+^-binding loop, and another Ca^2+^-binding loop reported at the end C-terminal of S100A14 [190,207,208,209,210,211,212].

#### 3.3.6. S100A16/S100 Calcium-Binding Protein A16

S100A16/S100F is a 10–12 kDa protein with the EF-hand Ca^2+^ binding motif significantly regulated in lung, ovarian, prostate, and breast cancers [140,213,214]. S100A16 mRNA has been found in a variety of tissues, including the brain, as astrocyte-specific glioblastoma cells, where it has been shown to accumulate in nucleoli and translocate to the cytosol in response to Ca^2+^ stimulation, implying that it may perform a role in ribonucleoprotein complex processing, gene silencing, or cell cycle development. The identified nuclear localization signal is absent in S100A16, as it is in other members of the S100 family. Phosphorylated S100A16 protein has been discovered in the nucleoli of HeLa cells, indicating that phosphorylation of S100A16 may play a role in nuclear translocation. S100A16 binds a single Ca^2+^ monomer at each atom, and it also binds Zn^2+^. However, the Ca^2+^ and Zn^2+^ binding sites are at distinct sites [215,216,217,218]. S100A16 expressions, mutually with S100A14, have been linked to a dangerous condition in patients with breast cancer; both proteins, in cooperation, can increase the aggressive activity of breast cancer cells via cytoskeleton functions. In MCF-7, high S100A16 accumulation supports epithelial–mesenchymal transition via the Notch-1 pathway, whereas low accumulation in oral squamous cell carcinoma patients is associated with deficient progenesis. It has been shown that the expression S100A16 in various malignancies is highly correlated with high prognostic outcomes. S100A16 in comparison to S100A14, in single cooperation in oral squamous cell carcinoma, indicated that S100A16 may serve the same purpose as S100A14. S100A14 is a prominent ligand of RAGE signaling pathways, and S100A16 is the most associated protein, implying that S100A16 has a role in tumor invasion and development [218,219,220,221,222]. Two common cell signaling pathways through serine/threonine kinases show S100A16 in prostate malignancy: MAPK/ERK and PI3K/AKT (Figure 1A) [223,224]. Consequently, with the suppression of p53 expression, S100A16 has been shown to promote metastasis. The high expression of S100A16, assumed in the relocation, invasion, and proliferation in human prostate cancer cells, was discovered in vitro [225].

Interestingly, the S100 family proteins have two sites for Ca^+^ binding, one at the EF-hand C-terminal and the other at the S100 short N-terminal. S100 family proteins have highly conserved Ca^+^ ligand amino acids at 1, 3, 5, 7, and 12 locations, which generate an enhanced attraction for metals. The backbone oxygen atoms of the residues at the positions of 1, 4, 6, and 9, as well as two side-chain oxygen atoms of the residues at the 14 locations, which is generally glutamate, are placed at the position of the 14th residue. However, due to the absence of glutamate at the comparable location S100A16, the Ca^+^ binding affinity is reduced by the S100A16 shortage of glutamate by the side of this site. In addition, the N-terminal has 15 amino acids rather than 14, which is unique to S100A16. Dimerization of Apo S100A16 with Ca^+^-loaded S100A16 generally occurs across communications between helices I, I_0_, IV, and IV_0_, which is responsible for forming an X-type helix package. Trp80 and Ile83, two hydrophobic amino acids in helix IV_0_, form many contacts with Leu8, Val12, and Leu15 in helix I_0_, as well as Trp80 and Ile83 in helix IV_0_ of the other subunit. Amino acids at positions Glu45, Leu46, His48, and Met49, in the hinge loop between helices II and III, form connections with amino acids towards the N-terminus of the subsequent subunit of helix I_0_. The connections in a dimeric arrangement of S100A16 align helices I and IV in opposing orders to helices I_0_ and IV_0_, respectively [226].

#### 3.3.7. S100B/S100 Calcium-Binding Protein B

S100B is a 10.7 kDa protein, and its expression has been reported in many disorders, including neurological diseases and cancer. High S100B absorptions continue to be used as a biomarker for malignant melanoma, with the increasing amount of S100B indicating cancer progression. High expression of S100B is still linked to unfavorable liver metastases and early deterioration in colorectal cancer. On other hand, high expression of S100B reveals ovarian malignancy-associated utilizing primary cancers. By activating NK-кB, S100B ligation with RAGE improves cell survival. S100B, a RAGE ligand that promotes tumorigenesis by promoting IL-6 expression and STAT-3 activation, is abundantly expressed in gliomas [227,228,229,230,231,232,233,234,235]. Based on the cell nature, RAGE, in conjugation with S100B and S100B, can activate STAT-3 through phosphorylation. S100B ligation with RAGE, a primary controller designed for downstream Akt-1 and STAT-3 activation, has been observed (Figure 1A). The results indicated that the C6 glioma-conditioned medium can promote the malignant transformation of mesenchymal stem cells and that this transformation is mediated by S1001B interaction with RAGE [120,236,237]. S100B has received much attention because of its binding to p53, a tumor suppressor gene in cancer that lowers the p53 protein level, perhaps hindering wild-type p53 malignant development [238,239,240,241,242,243]. S100B interaction with p53 causes Ca^+^-dependent progression; nevertheless, S100B ligation with Ca^+^ causes conformational rearrangement, causing several hydrophobic amino acids to become solvent-exposed. As a result, an interaction flanked by two associate proteins has been identified in rat S100B in multipart, using the C-terminal negative regulating domain, which comprises the amino acids in helices III and IV, and the S100B hinge loop [244,245]. Ca^2+^ binding reveals a significant hydrophobic grove near helix III on S100B. Homodimeric S100B binds two Ca^2+^, one at the position of the hinge-connected C-terminal and another at the site of the N-terminal EF-hand, indicating that many ligands result in RAGE-mediated signal transduction [38,39,246,247]. S100B can bind to Zn^2+^, Cu^2+^, and Ca^2+^; therefore, Zn^2+^ interaction with S100B increases the attraction, as mentioned above, to Ca^2+^, particularly in the peptide and protein function [38,39,246,247,248,249]. S100B ligation, in combination with RAGE activation, promotes oxidative stress through the activation of ERK1/2, p38, and JNK MAP kinase pathways [143,250,251]. S100B has a 12 amino-acid loop with acidic amino acids required for Ca^2+^ organization at Asp61 on the X-axis, Asp65 on the Y-axis, and Glu72 on the Z-axis, which has many homologous sequences in the S100 family Ca^2+^ binding EF-hand ligand. Helix III, which closely follows in the loop in human S100B, causes a conformational change in the C-terminal loop with Ca^2+^ binding, resulting in a redirection of helix III involving helices II and IV, as seen in rat apo S100B [210,211,252].

#### 3.3.8. S100P/S100 Calcium-Binding Protein P

**Figure 4:**
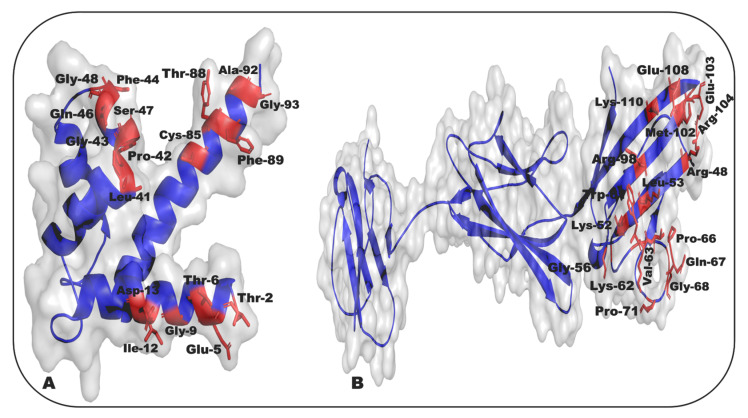
3D depictions of S100P and RAGE interacting residues: (**A**) S100P residues that interact with RAGE; and (**B**) residues found on the RAGE V-segment that interact with S100P.

S100P, a RAGE ligand, has been found in prostate, pancreatic, lung, breast, and colon cancers [253,254,255,256,257]. The interaction of RAGE with S100P stimulates major signaling pathways, including ERK1/2, NF-κB, and JAK/STAT, which increased S100P levels in colon cancers linked to metastasis (Figure 1A) [30,256,258,259,260]. S100P is highly expressed in pancreatic tumors, where it regulates multiple intracellular and extracellular processes, including cell proliferation, survival, treatment resistance, and diversity. S100P, on the other hand, includes the genesis of cancer and plays an important role in cancer progression. In vitro findings illustrate that the invasiveness is reduced by silencing S100P by employing siRNA, and in vivo findings illustrate a reduction in metastasis by silencing the S100P by using siRNA [31,256]. In vitro studies show that S100P is expressed in human colon cancer and stimulates cell growth, cell relocation, ERK phosphorylation, and NF-κB activation; these activities were mediated through RAGE [211]. S100P includes multiple α-helical concerns, with four systematic α-helical segments inserted at Glu3 to Ser19, Lys30 to Glu40, Ala53 to Ala63, Glu71 to His86, and another α-helical segment introduced in the linker region at Phe44 to Gly48. The Ca^2+^-binding loop I, on the other hand, attaches to helices I, one at Glu3 to Ser19 and the other at Lys30 to Glu40, forming the N-terminal EF-hand, whereas helices III at Ala53 to Ala63 and helices IV at Phe71 to His86 are linked via the Ca^2+^-binding loop II, forming the C-terminal EF-hand. The Ca^2+^-binding loop forms the N-terminal EF-Hand I attaching to helices I, one at Glu3 to Ser19 and the other at Lys30 to Glu40, whereas the C-terminal EF-hand is formed by the Ca^2+^-binding loop II connecting helices III at Ala53 to Ala63 and helices IV at Phe71 to His86. There appears to be no change in preceding Ca^2+^ binding in the S100 family, but there is a change in the relative position of loop I to helices I and II. Phe44 to Gly48 participate in a short α-helix that connects with two Ca^2+^ binding domains at Leu41 to Asp52 [261,262]. The hydrophobic surface of S100P at Tyr88 and Phe89 shows a substantial feature in the recognition of a target protein compared to other S100 family proteins, which play a crucial part in the acknowledgment of target proteins. S100P reveals the central linker region at sites 43 to 49, the amino acid contained at sites 83 to 94 in helix IV of the first monomer, and the 2nd to 14 amino acid of helix I, which is another monomer that indicates a prolonged surface binding directed at the RAGE V-segment. S100P hydrophobic residues are nonpolar amino acids of S100P, such as Ala92, Gly93, Phe89, Tyr88, Gly9, Ile12, Met8, Met1, Phe44, Gly43, Pro42, Leu41, and Gly48, that bind on the V-segment of the RAGE. The charge–charge interaction involving the acidic amino acids at the position of Glu5 and Asp13 represents the V-segment of RAGE to S100P, which may contribute to the maintenance of the complexes. Thr6, Thr2, Ser47, Gln46, and Cys85, as well as neutral and polar amino acids, make non-hydrophobic interactions with the RAGE V-segment. Amino acids on the RAGE V-segment that interact with the S100P ligand include the basic amino acids Arg48, Lys52, Lys62, Arg98, Arg104, and Lys110, which are responsible for the inter-spread of basic patches, as well as amino acids such as Leu53, Trp61, Val63, Pro66, Gly68, Gly56, Met102, and Pro71 as shown in Figure 4A,B [263,264].

### 3.4. Adhesion Molecules

Cell adhesion molecules are life-threatening in tumor growth and are classified into several functions and classifications. Cell adhesion molecules of various classes and roles can collaborate and temper the signaling purpose of receptor tyrosine kinases and soluble proteins in the extracellular matrix, accompanied by cadherins, integrins, immunoglobulin family CAMs, and CD44 [265]. They play a crucial role in cancer growth and metastasis in pancreatic cancer, tumorigenic cancer stem cells, melanoma, breast, lung, and oral cancer. [266,267,268,269]. Although RAGE has been suggested to maintain lung homeostasis by arbitrating cell adhesion molecules, the ERM protein family (ezrin, radixin, and moesin) has a strong cross-link between the cytoskeleton and the plasma membrane. It can interact with transmembrane proteins and the cytoskeleton, forming structural linkages that help to improve the cell cortex and control signal transmission pathways. According to a new study, the interaction of RAGE with ERM appears to be essential in regulating EMT-related structural alterations in alveolar epithelial cells. In pro-inflammatory cytokines that support EMT of human alveolar epithelial cells, ERM interaction with RAGE shows important stages in the rearrangement of the actin cytoskeleton [270]. RAGE shares substantial structural features with adhesion molecules such as ALCAM (CD166), BCAM (CD239), and MCAM (CD146, Mel-Cam, Muc18, and S-Endo1), according to recent findings [271]. MCAM has been linked to advancing melanoma, prostate cancer, and breast cancer. Members of the IgSF (immunoglobulin superfamily) association have been identified as biomarkers for cancer development.

Similarly, IgSF associates such as L1CAM, NCAM, PECAM-1, ALCAM, and ICAM-1 are associated with metastatic development in melanoma, glioma, breast, ovarian, endometrial, prostate, and colon cancer [272,273,274,275,276]. In colorectal cancer, cell adhesion molecules play an important role in determining the prognosis of this common disease. However, studies on adhesion molecule interactions with the RAGE axis may play a role in cancer progression and metastasis, and the machinery that causes the most significant reorganizations of the submembrane cytoskeleton is poorly understood, which could be a therapeutic target for cancer.

### 3.5. Complement Component

The complement component is a danger-sensing organization for external pathogens and endogenous modified characters. Although there is no conclusive proof, the complement component has a function in tumor resistance, according to available studies. The complement component, on the other hand, appears to be more effectively induced in tumor cells and their non-malignant specimens [277]. Even though each tumor has its unique profile of antigens and complements, the fascinating categorization of membrane components responsible for complement activation, and their pathways, is unclear. Tumors employ a variety of inhibitory mechanisms, including the articulation of CD35, CD46, CD55, and CD59 (membrane-bound proteins) with factor H or factor-H similar proteins, factor I, and C4b-binding protein, to alter complement activation (soluble regulatory proteins) [278,279,280,281]. Both complement stimulation results, anaphylatoxins C3a and C5a, can maintain chronic inflammation, promote an immunosuppressive microenvironment, induce angiogenesis, and increase cancer cell motility and spreading potential [282]. The complement component, which functions as a crucial character in both adaptive and innate immune systems, can work with the activator of immunoglobulin and non-immunoglobulin complement systems. By collaborating with a wide range of surface molecules, C1q plays a supervisory function in cellular processes [283]. C1q also helps other dangerous immune functions, such as allowing the apoptotic to be activated and modulating cellular activities within adaptive immune responses, and an integrin b2, also known as MAC-1, can bind to the collagen domain of C1q, while RAGE, as a receptor for Mac-1, may generate a complex structure that can enhance the binding affinity [284,285,286,287]. RAGE acts as a receptor for the globular domain complement component C1q, which improves C1q-mediated phagocytosis by allowing for critical interactions in response to RAGE and C1q [288].

### 3.6. Advanced Lipoxidation end Products (ALEs)

Advanced lipoxidation end products (ALEs) are a collection of adducts and cross-links formed by the nonenzymatic response of reactive carbonyl species (RCS) produced by lipid peroxidation, lipid breakdown by nucleophilic sites of proteins (primarily Cys, Lys, His, and Arg residues), amino phospholipids, and DNA [289]. Lipoxidation of a protein is a nonenzymatic post-translational modification that occurs when reactive lipid molecules are covalently incorporated into proteins. Studies have correlated protein lipoxidation to cancer, neurodegeneration, and atherosclerosis [290].

Lipoxidation is a well-known process between electrophilic carbonyl species and certain proteins, which is characterized by the oxidation of lipids and, in some situations, the alteration of protein characteristics. Studies on lipoxidation have revealed the impact of cancer growth on tumor cells, the change of immune elements, and subsequent immune response stress. Protein adducts disrupt a variety of proteins in cancer that can activate various types of machinery, such as cell division, differentiation, and death. In in vivo findings on the human colon, adenocarcinoma showws a reduced appearance of TGFb-1 and a scarcity of 4-HNE protein adducts, implying that neoplastic evolution is healthier under these conditions. Meanwhile, TGFb-1 and 4-HNE collaborate to promote colon cell apoptosis by activating the JNK pathway [291,292]. At normal conditions, the adducted residues are in their neutral configuration, a proportional change in their ionization state that weakens the ionic bridges and makes nearby anionic residues more approachable and accessible to relieve ion pairs with the RAGE-positive residues. The ability of ALEs to interact with RAGE protein is mostly determined by the type of the adduct and the modification site [293].

### 3.7. Lipopolysaccharide

Lipopolysaccharide is a bacterial antigen that plays a vital role in cancer progression by encouraging the release of IL-8 and TGF-1, which promotes the growth and invasion of liver cancer cells, colorectal cancer cells, and leukemia cells [294,295,296,297]. TLR4 is a major LPS receptor, and RAGE can interact with LPS and cause pro-inflammatory signaling [298]. TLR4 is an important LPS receptor, and RAGE can interact with LPS and cause pro-inflammatory signaling [298,299]. According to the latest in vitro studies in breast cancer cells, LPS increases the expression of S100A7, which inhibits TLR4 and promotes RAGE accumulation in breast carcinogenesis. In vitro, treatment of LPS on an S100A7 overexcited in breast cancer cell line (MDA-MB-231), and in vivo treatment of MVT-1 (mouse mammary tumor cell line) with mS100A7/A15 revealed enhanced RAGE manifestation in a dose-dependent and time-dependent manner [75,300]. Another study found that LPS-induced inflammation of normal cervical epithelial cells promotes the malignant transformation of the cell. Additionally, the LPS may promote the proliferation and invasion of normal epithelial cells; the accumulation of IL-1, IL-6, and TNF were promoted in cervical epithelial cell lines, whereas RAGE inhibitors altered the LPS [301].

## 4. RAGE-Specific Signaling in Cancer Progression

RAGE–AGE interaction enhances the activation of the PI3K, oncogenic Ras, PKC, and Rho/GTPase partners (Cdc-42 and Rac-1) signaling pathways, which is mandatory for cell survival, stress responses, apoptosis, the release of growth factors, proinflammatory cytokines, and motility (Table 1) [21,207,301,302]. A protein initiates the release of IL-1, called the nucleotide-binding oligomerization domain-like receptor. Whereas IL-1, on the other hand, stimulates the nuclear translocation of HMGB-1 to the cytoplasm. Extracellularly, HMGB-1 can interact with RAGE and TLRs to trigger the release of proinflammatory cytokines, such as NF-κB, and elicit physiological responses. HMGB-1 tempted signaling through surface receptors, such as receptors for advanced glycation end-product and toll-like receptors, can discharge pro-inflammatory cytokines and chemokines, which include detrimental inflammatory responses, according to in vitro and in vivo investigations [19,50,91,92,106,111,303,304]. Intracellular signaling of HMGB-1 via RAGE and TLR activates many signaling pathways, including MAP kinases and JNK, as well as NF-κB translocation and inflammation [126,305]. In CRC cell lines, S100A4 interacts extracellularly with RAGE and impacts cell motility and metastasis, priming MAPK/ERK hyperactivation and hypoxia signaling (Figure 2E) [127]. Previously, in vitro studies suggested that S100A4 interaction with RAGE are involved in various cancers and activate several signaling pathways, including NF-κB upregulation in human osteosarcoma, human melanoma, and human pancreatic cancer, and ERK or Cdc42 upregulation in human colorectal carcinoma and thyroid cancer [127,203,299,305,306]. The combination of S100A6 and RAGE in vivo has been shown to produce nasopharyngeal cancer by upregulating p38 [170]. RAGE and S100A7 interaction were discovered to be involved in breast cancer and human cervical cancer through upregulation of ERK, MMP9, and NF-κB in vivo and in vitro investigations (Figure 2E) [171,182,307]. The upregulation of NF-κB, p38, ERK1/2, SAPK/JNK and the upregulation of p38, p-erk1/2 in human hepatocellular carcinoma can cause human breast cancer, prostate cancer, colon tumor, oral, esophageal tumor, and squamous carcinoma.

In vitro and in vivo findings of S100A8/9 interaction with RAGE can cause human breast cancer, human prostate cancer, colon tumor, and oral and esophageal tumors [225,308,309,310,311]. The overexpression of ERK1/2, NF-κB, and Akt, as well as the downregulation of p21 and p27, have been found to have a role in diverse malignancies, such as esophageal squamous cell carcinoma and human prostate cancer in vitro [235,312]. In vitro data on the interaction of RAGE with S100B/P suggest a role in ovarian cancer, glioma (C6), and human colon adenocarcinoma by downregulating p53 and upregulating Akt1, STAT3, and in nasopharyngeal carcinoma, colon cancer, and pancreatic cancer by upregulating NF-κB, ERK1/2, MMP2, and MMP9 [256,298,299,313,314,315,316]. According to recent in vitro studies in breast cancer cells, LPS increases the expression of S100A7, which inhibits TLR4 and promotes RAGE accumulation in breast carcinogenesis. In invasive breast cancer cells, RAGE interaction with S100A7 mediates oncogenic response; in vitro, LPS treatment of S100A7 rose in a breast cancer cell line (MDA-MB-231). In vivo treatment of the mouse mammary tumor cell line (MVT-1) with mS100A7/A15 revealed enhanced RAGE expression in a dose- and time-dependent manner [23,112].

## 5. Anti-RAGE Therapeutics in Cancer Management

**Figure 5:**
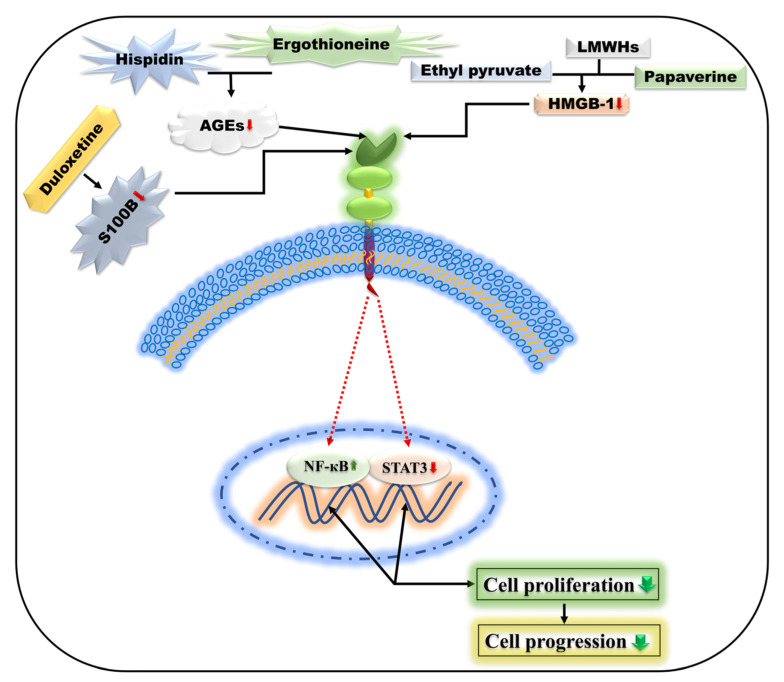
RAGE inhibitors such as duloxetine, hispidin, ergothioneine, ethyl pyruvate low-molecular-weight heparins (LMWHs), and papaverine diminish the formation of AGEs, S100B, and HMGB1 and have an impact on downstream signaling (red arrow in downward direction). However, these inhibitors upregulate NF-κB (green arrow upward direction) and downregulate STAT3 (red arrow downward direction) which are primarily involved in the regulation of cancer progression. Moreover, another green arrow indicates the suppression of cancer progression (green arrow in downward direction).

In vitro experiments with S100 proteins and HMGB-1 in mutual ligation with RAGE stimulate HMGB-1 ligation on RAGE. Likewise, via S100P, the first 40 amino acids of HMGB-1, the HMGB-1 derivative peptide, can prevent the ligation and activation of RAGE. RAGE inhibitor peptide sequences occur at residues 32–42 on S100P in the same region as many other S100 family members. RAGE inhibitor peptides can disrupt the connection between S100P, HMGB-1, and S100A4 on RAGE as shown in Figure 5 (Table 2) [31,317,318]. The impact of RAGE inhibitors on various cancers is described further down. RAGE inhibitors have been investigated for their potential to inhibit cancer progression. Some of these are described below.

### 5.1. siRNA Inhibitor

RAGE has been identified as an oncogenic protein that promotes the development and spread of various malignancies. However, the migratory and invasive abilities of NSCLC cells are reduced when siRNA inhibits RAGE. RAGE is also involved in EMT, reducing RAGE and initiating transmutation from mesenchymal to epithelial signals in cell lines. Moreover, we found RAGE to be a factor in epithelial–mesenchymal transition (EMT), with RAGE deficiency resulting in the transmutation of mesenchymal to epithelial markers in cell lines. According to in vivo data, siRNA can decrease RAGE and tumor growth and proliferation [320,321,322]. In NSCLC cells, siRNA-suppressed RAGE inhibits PI3K/AKT activity, impacts downstream mTOR/p70S6K signaling, and reduces the phosphorylation initiation of KRAS and RAF-1 at the same time. As a result, RAGE may have a role in NSCLC growth, metastasis, and other malignancies [322] and siRNA targeting of RAGE might be useful as a therapeutic approach for NSCLC.

### 5.2. shRNA Inhibitor

RAGE accumulation is associated with increased microvessel efficiency in colorectal tissue samples, and the familiar method can reduce VEGF expression by silencing RAGE expression, implying that RAGE plays a role in VEGF expression and blood vessel formation in colorectal cancer. Furthermore, the VEGF gene promoter’s convenient domain has significant GC-rich motifs that may be modulated by transcriptional factor explicitness (SP1) [323,324]. According to the findings, silencing RAGE affects a variety of stages of colorectal cancer angiogenesis, including an SP1 decrease and VEGF articulation [325,326].

### 5.3. RBGO1 and ADC

In contrast to RAGE, RGBO1 is a monoclonal antibody. KLH (keyhole limpet hemocyanin) linked RAGE was injected into mice, and the KLH coupled peptides corresponding to amino acid residues 198–217 or 327–344 of the RAGE protein. Replicas were selected based on a positive ELISA screen utilizing BSA (bovine serum albumin) linked peptides. RGBO1 is a clone of the full-length RAGE protein [145]. In vivo efficacy was determined by injecting RBGO1 ADC (3 mg/kg) or MCF (45 g/kg) into female nude mice with 5mm HEC1A xenograft tumors on a two-time weekly basis for four weeks. As a result, whereas RGBO1 therapy alone had no significant effect on cell survival, RGBO1 treatment on MCF was determined to be effective.

Furthermore, RBGO1 ADC is effective in vivo at a 7.4 µg/ml concentration. The RAGE importance on RBGO1 ADC was significantly more potent than the same ADC focus on HER2. RAGE-directed efficacy of ADCs is based on V-domain binding, and RGBO1 ADC is 200-fold more effective than cytotoxic drug therapy alone [13]. These findings suggest that using RAGE-targeting RGBO1 ADCs as a treatment for endometrial cancer is highly effective.

### 5.4. Sorafenib

Sorafenib, a kinase inhibitor, has been approved to treat early-stage kidney cancer, advanced liver cancer, and thyroid carcinoma. RAGE is overexpressed in both tumor and stromal cells; however, RAGE accumulation is more prominent in tumors, and RAGE targets in HCC cell lines can enhance autophagy [327]. RAGE activation by the AMPK/mTOR pathway triggers autophagy. Furthermore, sorafenib promoted apoptosis by modifying AMPK activity and preventing RAGE. By regulating autophagy, RAGE promotes HCC proliferation and sorafenib resistance. An in vitro investigation identified HCC and sorafenib resistance as probable causes [327].

### 5.5. Duloxetine

Duloxetine, commonly known as serotonin/noradrenaline, is a reuptake inhibitor (RI) used to treat depression and neuropathic pain. It has also been employed for the treatment of chemotherapy-induced acentric neuropathy [321,325]. Gao et al. examined GL261 cell lines of mouse glioma cells using duloxetine in a dose-dependent manner (30 mg/kg) by way of oral feeding for 14 days and concluded that duloxetine has the potential to suppress S100B production as well as the inhibition of GL261 glioma cell proliferation within the cranium [113].

### 5.6. Heparin

In various studies, an anti-thrombotic medicine, achieving the perfect anti-coagulant, was critically employed for modern hematology and oncology with the affected patient, who was at the greatest risk of bleeding and circulatory thromboembolism through RAGE of an NF-κB dependent on luciferase reporter assay and the human fibrosarcoma cell line (HT1080); heparin decreased the HMGB1 level and encouraged the activation of NF-κB (Figure 5). Heparin, but not HT1080mock or HT1080dnRAGE cells, suppressed the relocation, invasion, tumor formation, and lung metastasis of the human fibrosarcoma cell line (HT1080RAGE cells) [23]. According to reported data, heparin had therapeutic potential in individuals with specific types of malignant tumors, such as chondroitin sulfate and heparan sulfate-directed RAGE, suggesting that lung metastasis was reduced [110,326].

### 5.7. S100P-Derived RAGE Inhibitor

The HMGB-1 antagonist peptide sequence was compared to the S100P sequence and a resemblance was found in amino acid sequence and pattern between the first 40 residues of S100P and the 33 residues of RAGE-obligatory peptide derived from HMGB-1. The discovery of this likeness resulted in the modeling of three peptides from the S100P sequence, each of which was 95% pure. The peptides were designed with the amino terminus by amidation, and the carboxyl terminus by acetylation to improve their consistency. A RAGE inhibitor derived from S100P can prevent the adherence of various RAGE ligands involved in inflammation and cancer. In vivo data revealed that the S100P-derived inhibitor may have therapeutic potential against glioma and pancreatic cancer and may inhibit a variety of RAGE ligands, implying that it could be useful in additional RAGE-related diseases [3,85].

### 5.8. FPS-ZM1 and LY294002

RAGE overexpression stimulates cervical cancer cell proliferation and increases the appearance of proliferating cell nuclear antigens. Meanwhile, RAGE overexpression inhibited cells, resulting in apoptosis suppression, a reduction in Bax/Bcl-2 levels, and activation of PI3K/AKT. SiHa cell capability and PCNA appearance were decreased by FPS-ZM1, a RAGE inhibitor, which also increased cell death and Bax/Bcl-2 levels. The PI3K inhibitor LY294002 decreased PI3K and AKT activation and RAGE accumulation, promoting cell proliferation and death [3]. It has a wide range of RAGE ligands, suggesting that it may be effective in other RAGE-related disorders.

### 5.9. Gefitinib

HMGB1 has a key function in autophagy stimulation in vital carcinogenesis, and it is mostly found in the cytoplasm of human gastric cancer cells with cell death and survival supervisory body. Gefitinib is an EGFR inhibitor, and HMGB1 release from tumor cells treated with gefitinib promotes autophagy, interacts with RAGE, and induces ERK1/2 signaling in gastric cancer cells. HMGB1 inhibitors, such as gefitinib, and RAGE inhibitors might play an essential role in preventing cancer regrowth, as suggested by HMGB1-related autophagy in chemotherapy [41].

### 5.10. Metformin

Metformin use has been linked to duction in the occurrence and mortality of breast cancer in diabetics patient. AGEs appear to have accelerated MCF-7 cell proliferation, which was inhibited by 0.01mM or 0.1mM metformin or anti-RAGE antibodies. Metformin, at a dosage of 0.01 mM, also suppressed the AGE-induced upregulation of RAGE and VEGF mRNA levels in MCF-7 cells. In AGEs-exposed MCF-7 cells, compound C, an AMP-triggered protein, appeared to block the growth inhibition of metformin, RAGE, and VEGF effects. As reported, metformin may limit AGE-induced growth and VEGF expression in MCF-7 breast cancer cells by RAGE expression via the AMP-triggered protein kinase pathway. As a result, metformin may protect diabetic individuals against breast cancer expansion by inhibiting the AGEs–RAGE axis [328,329,330,331,332,333].

### 5.11. RAP (RAGE Antagonist Peptide)

*In vivo* and in vitro research reveals that RAP inhibitor inhibits the ligation of S100A4, S100P, and HMGB-1 with RAGE at micromolar concentrations. The efficiency of the ligands to promote RAGE activation of NF-κB in cancer cells is hampered by the presence of a RAP inhibitor. Systemic in vivo study of RAP significantly reduced the growth and spread of pancreatic and glioma tumors. The RAP has the potential to explore RAGE issues and cure RAGE-related disorders in vivo [304].

### 5.12. Cromolyn

Cromolyn is an antiallergen, and its study revealed that it inhibits S100P. Cromolyn is a RAGE inhibitor that reduces cancer growth, endurance, and invasiveness in vitro by inhibiting RAGE interaction with S100P. Cromolyn inhibited the NF-κB pathway in pancreatic cancer with endogenic S100P in vitro and in vivo. In mouse models, studies show that the processes clarify the pragmatic suppression of tumor development and cromolyn’s capacity to increase gemcitabine’s efficacy in eradicating pancreatic cancer cells [315].

### 5.13. Ethyl Pyruvate

An ethyl ester of pyruvic acid effectively inhibits HMGB1 in treating inflammatory diseases and malignancies [334,335,336]. The effectiveness of ethyl pyruvate on human malignant mesothelioma cells and tumor formation has been demonstrated in vivo investigations. Because ethyl pyruvate successfully decreased RAGE accumulation and the activation of NF-κB, research has discovered that it has a significant role in reducing HMGB1 release in malignant mesothelioma cells. Furthermore, ethyl pyruvate reduced the HMGB1 serum scale in vivo and suppressed the growth of malignant mesothelioma xenografts [336]. Moreover, an in vitro study found that ethyl pyruvate can inhibit non-small cell lung cancer growth and progression by inducing apoptosis via the HMGB1-RAGE axis and the NF-κB/STAT3 pathway (Figure 5) [337]. Consequently, ethyl pyruvate has been identified as a promising treatment for malignant mesothelioma, with the ability to reduce the progression of NSCLC.

### 5.14. Papaverine

A non-narcotic opium alkaloid derived from the plant *Papaver somniferum*. Contrary to multiple tumor cells, papaverine demonstrated discriminating anticancer effects [338,339]. An in vitro experiment was performed to test the anti-RAGE result of papaverine, improved using the structure-based pharmacological technique known as a conversion-to-small-molecules-via-optimized-peptide strategy (COSMOS), in human fibrosarcoma cell lines (HT1080). It was revealed utilizing RAGE or dnRAGE expressing (dominant-negative) HT1080 cell lines that papaverine inhibited RAGE-dependent human fibrosarcoma cell propagation, relocation, and invasion in a dose-dependent manner by significant suppression of RAGE-dependent NF-κB determined by HMGB1 (Figure 5) [339]. Furthermore, papaverine inhibited cell proliferation in human glioblastoma (U87MG and T98G) cell lines via downregulating HMGB1 and RAGE [339]. Therefore, papaverine may inhibit RAGE and can be safeguarded as an encouraging anticancer medication.

### 5.15. Hispidin and Ergothioneine

Hispidin is a polyphenol derived from the fungus *Phellinus linteus* that can be used as a major medication due to its biological applicability as an antioxidant and anticancer. Hispidin may be a novel gemcitabine chemosensitizer and a cooperative representative that can shift the gemcitabine therapeutic indication to pancreatic cancer treatment. Furthermore, hispidin induces apoptosis in colon cancer cells via a class of reactive oxygen species. Fungi and bacteria make ergothioneine, a thiol molecule. A mixture of 2 µM ergothioneine and hispidin reduces the development of AGEs, RAGE expression, and the activation of NF-κB in a PC12 cell line from rat pheochromocytomata (Figure 5) [340,341,342,343]. The AGEs–RAGE axis-associated stimulation of carcinogenesis was minimized in collaboration with ergothioneine and hispidin.

## 6. Conclusions

We reviewed RAGE and its ligands, as well as various inhibitors that can provide a platform for researchers and clinicians to develop a novel strategy for tackling numerous types of cancers, considering that RAGE is a cell surface receptor known to interact with a wide range of ligands. Multiple small RAGE inhibitors were explored in this review for their ability to inhibit RAGE in various malignancies. The current research shows that RAGE and its interaction with ligands increase the efficiency of antiapoptotic proteins while diminishing the effectiveness of proapoptotic proteins in promoting cancer cell proliferation, invasion, and angiogenesis. In the initial sections of this review, we described the role of RAGE ligands in cancer progression, particularly AGEs, HMGB1, S100 proteins, adhesion molecules, complement components, ALEs, and lipopolysaccharides, which play important roles in cancer biology in conjunction with RAGE. In the later sections, we focused on various anti-RAGE therapeutics, particularly mAbs, peptides, and small molecules, for managing cancer metastasis. The utilization of existing and newly discovered RAGE inhibitors for cancer prognosis might reduce the burden of cancer. Additionally, several existing RAGE inhibitors, acting as anticancer drugs, which could be beneficial in integrated treatment strategies, were also presented.

## Figures and Tables

**Table 1:** RAGE–ligand interaction in different malignancies.

S. No.	RAGE–Ligand Interaction	Cancer Type	In Vitro/In Vivo Study	References
1.	RAGE–AGEs	Human oral cancer, breast cancer, prostate carcinoma cells	In vitro	[2,3,4,5]
2.	RAGE–HMGB1	Gastric cancer, colon cancer, breast cancer, melanoma, prostate cancer, hepatocellular carcinoma	In vitro	[2,6,7,8,9,10,11]
3.	RAGE–S100A4	Human colorectal cancer, melanoma, human osteosarcoma, human pancreatic cancer, thyroid cancer (human specimens)	In vitro/In vivo	[12,13,14,15,16]
4.	RAGE–S100A6	Nasopharyngeal carcinoma	In vitro	[10]
5.	RAGE–S100A7	Breast cancer, human cervical cancer	In vitro/In vivo	[17,18]
6.	RAGE–S100A8	Human breast cancer, human prostate cancer, colon tumor, oral-esophageal tumor, squamous cell carcinoma	In vitro/In vivo	[19,20,21,22,23]
7.	RAGE–S100A9	Human hepatocellular carcinoma	In vitro	[24]
8.	RAGE–S100A14	Esophageal squamous cell carcinoma	In vitro	[25]
9.	RAGE–S100A16	Human prostate cancer	In vitro	[26]
10.	RAGE–S100B	Ovarian cancer, glioma (C6), human colon adenocarcinoma	In vitro	[27,28,29,30]
11.	RAGE–S100P	Nasopharyngeal carcinoma, colon cancer, pancreatic cancer,	In vitro/In vivo	[16,31,32]
12.	RAGE–LPS	Liver cancer cells, colorectal cancer, and leukemia cells	In vitro/In vivo	[33,34]

**Table 2:** RAGE inhibitors are designed for diverse human malignancies.

S. No.	RAGE Inhibitors	In Vitro/In Vivo Findings	RAGE Pathways	Studies	Cancer Types	References
1.	RGBO1	In vitro/In vivo	AKT, BCL2, and cyclin D1	HEC1A, HEC1B, HEC50/Murine xenograft model	Endometrial cancer (EC)	[35,36]
2.	Sorafenib	In vitro/In vivo	AMPK/mTOR signaling	HepG2, HCCLM3, Huh7, Bel7402/xenografts and orthotopic nude mice models	Hepatocellular carcinoma (HCC)	[37]
3.	S100B inhibitor /K-Luc (*Duloxetine)*	In vitro/In vivo	STAT3	GL261/Murine model	Gliomas	[38]
4.	Sulfated Glycosaminoglycans (Chondroitin sulfate (CS), Heparan sulfate (HS))	In vivo	RAGE-mediated pathway	Male C57BL/6 mice	Lung metastasis	[39,319]
5.	S100P-derived inhibitor	In vitro/In vivo	via NF-κB	C6-glioma, MPanc-96 for pancreatic cancer/Murine model	Pancreatic tumors and Glioma tumor growth	[33]
6.	RAGE inhibitor FPS-ZM1/ PI3K inhibitor LY294002	In vitro/In vivo	PI3K/AKT	SiHa, CaSki, C33A, MS751/xenograft mouse model	Cervical squamous cell carcinoma (CSCC)	[40]
7.	Gefitinib	In vitro	ERK1/2 MAPK	GES-1	Gastric carcinoma cells	[41]
8.	Metformin/RAGE-Ab	In vitro	AMP-activated protein kinase	MCF-7	Breast cancer cells	[42]
9.	(RAP) Potent LRP1 antagonist	In vivo	NF-κB signaling	Nude mice	Pancreatic tumors, glioma tumor growth	[43]
10.	Cromolyn	In vivo	NF-κB signaling	Male CB17 SCID mice	Pancreatic cancer	[31]
11.	Ethyl pyruvate	In vitro/In vivo	NF-κB/STAT3 signaling	A549, H520, and PC-9 cell lines/orthotopic MM xenograft model	Human malignant mesothelioma/non-small cell lung cancer cells	[44]
12.	Papaverine	In vitro	NF-κB signaling	HT1080, U87MG, and T98G cell lines	Human fibrosarcoma, human glioblastoma	[45]
13.	Ergothioneine and Hispidin	In vitro	NF-κB signaling	PC12 cell lines	Rat pheochromocytoma	[46]
